# Supplementary material for: Hot spot prediction in protein-protein interactions by an ensemble system
Source: BMC Syst Biol. 2018 Dec 31;12(Suppl 9):132. doi: 10.1186/s12918-018-0665-8 (PMC6311905; doi:10.1186/s12918-018-0665-8)
Supplement: Supplementary file 2 — All relevant MATLAB code. A simple Matlab implement of our predictor. (ZIP 19,742 kb) [file 12918_2018_665_MOESM2_ESM.zip › Additional file 2/Read ASA/ASA Property/SKEMPI/2C0LA.txt]

 NetSurfP - Protein Surface Accessibility and Secondary Structure Predictions

  

|  |  |  |
| --- | --- | --- |
|  |  | NetSurfP - Protein Surface Accessibility and Secondary Structure Predictions  Technical University of Denmark |

  


---

```
# For publication of results, please cite:
# A generic method for assignment of reliability scores applied to solvent accessibility predictions.
# Bent Petersen, Thomas Nordahl Petersen, Pernille Andersen, Morten Nielsen and Claus Lundegaard
# BMC Structural Biology 2009, 9:51 doi:10.1186/1472-6807-9-51
#
# Column 1: Class assignment - B for buried or E for Exposed - Threshold: 25% exposure, but not based on RSA
# Column 2: Amino acid
# Column 3: Sequence name
# Column 4: Amino acid number
# Column 5: Relative Surface Accessibility - RSA
# Column 6: Absolute Surface Accessibility
# Column 7: Z-fit score for RSA prediction
# Column 8: Probability for Alpha-Helix
# Column 9: Probability for Beta-strand
# Column 10: Probability for Coil
E H  2C0LA                  1    0.631 114.779  -0.829   0.003   0.003   0.994
E P  2C0LA                  2    0.566  80.301   0.015   0.717   0.014   0.269
E Q  2C0LA                  3    0.423  75.601   0.086   0.802   0.014   0.185
B P  2C0LA                  4    0.161  22.789   0.809   0.938   0.007   0.055
B F  2C0LA                  5    0.198  39.819   0.016   0.938   0.007   0.055
E E  2C0LA                  6    0.464  81.113   0.067   0.938   0.007   0.055
B E  2C0LA                  7    0.216  37.823   1.619   0.938   0.007   0.055
B G  2C0LA                  8    0.023   1.810  -0.591   0.970   0.001   0.030
B L  2C0LA                  9    0.262  47.917   1.406   0.970   0.001   0.030
B R  2C0LA                 10    0.278  63.639   1.242   0.988   0.000   0.012
B R  2C0LA                 11    0.124  28.373   0.914   0.970   0.001   0.030
E L  2C0LA                 12    0.307  56.248   0.987   0.938   0.007   0.055
E Q  2C0LA                 13    0.654 116.822   1.001   0.879   0.010   0.111
E E  2C0LA                 14    0.479  83.751   0.504   0.321   0.003   0.675
E G  2C0LA                 15    0.609  47.936  -0.403   0.053   0.005   0.942
E D  2C0LA                 16    0.437  63.001   1.400   0.016   0.005   0.979
B L  2C0LA                 17    0.146  26.751   0.992   0.694   0.003   0.303
E P  2C0LA                 18    0.671  95.257   1.343   0.938   0.007   0.055
E N  2C0LA                 19    0.473  69.189   1.951   0.975   0.003   0.022
B A  2C0LA                 20    0.012   1.366   1.350   0.975   0.003   0.022
B V  2C0LA                 21    0.162  24.899   1.154   0.970   0.001   0.030
E L  2C0LA                 22    0.591 108.212   1.772   0.970   0.001   0.030
B L  2C0LA                 23    0.156  28.619   1.795   0.970   0.001   0.030
B F  2C0LA                 24    0.025   4.957   1.228   0.970   0.001   0.030
E E  2C0LA                 25    0.430  75.086   2.261   0.970   0.001   0.030
E A  2C0LA                 26    0.417  45.931   2.359   0.988   0.000   0.012
B A  2C0LA                 27    0.031   3.427   0.521   0.970   0.001   0.030
B V  2C0LA                 28    0.057   8.807   1.143   0.938   0.007   0.055
E Q  2C0LA                 29    0.651 116.197   1.859   0.879   0.010   0.111
E Q  2C0LA                 30    0.355  63.457   1.414   0.600   0.003   0.397
E D  2C0LA                 31    0.278  39.988   1.339   0.053   0.005   0.942
E P  2C0LA                 32    0.457  64.806  -0.601   0.053   0.005   0.942
E K  2C0LA                 33    0.699 143.784   0.218   0.016   0.005   0.979
B H  2C0LA                 34    0.174  31.596   0.542   0.018   0.019   0.964
B M  2C0LA                 35    0.257  51.366  -0.113   0.923   0.002   0.076
E E  2C0LA                 36    0.472  82.493   1.149   0.970   0.001   0.030
B A  2C0LA                 37    0.033   3.626   0.110   0.970   0.001   0.030
B W  2C0LA                 38    0.054  12.891   0.758   0.970   0.001   0.030
B Q  2C0LA                 39    0.174  31.130  -0.141   0.970   0.001   0.030
B Y  2C0LA                 40    0.166  35.517  -0.233   0.970   0.001   0.030
B L  2C0LA                 41    0.060  10.931   1.233   0.970   0.001   0.030
B G  2C0LA                 42    0.026   2.015  -0.966   0.970   0.001   0.030
B T  2C0LA                 43    0.184  25.562   1.121   0.970   0.001   0.030
B T  2C0LA                 44    0.029   3.953   0.642   0.988   0.000   0.012
B Q  2C0LA                 45    0.095  16.949   1.042   0.970   0.001   0.030
E A  2C0LA                 46    0.362  39.914   0.907   0.938   0.007   0.055
E E  2C0LA                 47    0.539  94.216   1.026   0.879   0.010   0.111
E N  2C0LA                 48    0.347  50.801   0.465   0.321   0.003   0.675
E E  2C0LA                 49    0.644 112.419  -0.169   0.053   0.005   0.942
E Q  2C0LA                 50    0.451  80.513   1.608   0.053   0.005   0.942
B E  2C0LA                 51    0.165  28.773   0.720   0.694   0.003   0.303
E L  2C0LA                 52    0.731 133.828   1.377   0.938   0.007   0.055
E L  2C0LA                 53    0.484  88.675   2.043   0.938   0.007   0.055
B A  2C0LA                 54    0.013   1.466   1.205   0.975   0.003   0.022
B I  2C0LA                 55    0.163  30.229   1.355   0.970   0.001   0.030
E S  2C0LA                 56    0.594  69.582   1.851   0.970   0.001   0.030
B A  2C0LA                 57    0.135  14.844   1.465   0.970   0.001   0.030
B L  2C0LA                 58    0.032   5.804   0.838   0.988   0.000   0.012
E R  2C0LA                 59    0.434  99.317   2.002   0.988   0.000   0.012
E R  2C0LA                 60    0.411  94.188   2.153   0.970   0.001   0.030
B C  2C0LA                 61    0.021   2.962   0.984   0.970   0.001   0.030
B L  2C0LA                 62    0.091  16.680   1.017   0.938   0.007   0.055
E E  2C0LA                 63    0.647 112.944   1.361   0.802   0.014   0.185
E L  2C0LA                 64    0.238  43.596   0.964   0.406   0.004   0.590
E K  2C0LA                 65    0.320  65.742   1.538   0.016   0.005   0.979
E P  2C0LA                 66    0.488  69.290  -0.358   0.053   0.005   0.942
E D  2C0LA                 67    0.684  98.636   0.080   0.016   0.005   0.979
B N  2C0LA                 68    0.167  24.449   0.404   0.109   0.005   0.886
B Q  2C0LA                 69    0.240  42.828  -0.229   0.923   0.002   0.076
E T  2C0LA                 70    0.492  68.171   1.271   0.970   0.001   0.030
B A  2C0LA                 71    0.033   3.681   0.074   0.970   0.001   0.030
B L  2C0LA                 72    0.066  12.048   1.041   0.970   0.001   0.030
B M  2C0LA                 73    0.173  34.557  -0.213   0.970   0.001   0.030
B A  2C0LA                 74    0.157  17.290  -0.634   0.970   0.001   0.030
B L  2C0LA                 75    0.067  12.268   0.801   0.970   0.001   0.030
B A  2C0LA                 76    0.028   3.097  -1.343   0.970   0.001   0.030
B V  2C0LA                 77    0.203  31.216   1.219   0.970   0.001   0.030
B S  2C0LA                 78    0.027   3.164   0.748   0.988   0.000   0.012
B F  2C0LA                 79    0.106  21.234   0.893   0.970   0.001   0.030
E T  2C0LA                 80    0.387  53.663   0.992   0.938   0.007   0.055
E N  2C0LA                 81    0.575  84.239   1.093   0.858   0.002   0.139
E E  2C0LA                 82    0.290  50.576   0.488   0.406   0.004   0.590
E S  2C0LA                 83    0.654  76.649  -0.084   0.109   0.005   0.886
E L  2C0LA                 84    0.408  74.741   1.457   0.109   0.005   0.886
B Q  2C0LA                 85    0.173  30.844   0.773   0.782   0.003   0.216
E R  2C0LA                 86    0.706 161.628   1.339   0.938   0.007   0.055
E Q  2C0LA                 87    0.491  87.746   1.944   0.975   0.003   0.022
B A  2C0LA                 88    0.012   1.355   1.361   0.970   0.001   0.030
B C  2C0LA                 89    0.167  23.447   1.452   0.970   0.001   0.030
E E  2C0LA                 90    0.587 102.566   1.875   0.970   0.001   0.030
B I  2C0LA                 91    0.140  25.881   1.460   0.970   0.001   0.030
B L  2C0LA                 92    0.026   4.797   1.247   0.988   0.000   0.012
E R  2C0LA                 93    0.443 101.516   2.134   0.988   0.000   0.012
E D  2C0LA                 94    0.408  58.865   2.292   0.988   0.000   0.012
B W  2C0LA                 95    0.029   7.023   0.641   0.970   0.001   0.030
B L  2C0LA                 96    0.083  15.179   1.216   0.938   0.007   0.055
E R  2C0LA                 97    0.644 147.430   1.452   0.879   0.010   0.111
E Y  2C0LA                 98    0.242  51.715   1.340   0.522   0.016   0.462
E T  2C0LA                 99    0.319  44.245   1.523   0.016   0.005   0.979
E P  2C0LA                100    0.556  78.953  -0.536   0.016   0.005   0.979
E A  2C0LA                101    0.715  78.749   0.478   0.016   0.005   0.979
B Y  2C0LA                102    0.150  31.991   0.681   0.109   0.005   0.886
B A  2C0LA                103    0.319  35.187  -0.601   0.923   0.002   0.076
E H  2C0LA                104    0.463  84.274   1.055   0.970   0.001   0.030
B L  2C0LA                105    0.034   6.189  -0.263   0.970   0.001   0.030
B V  2C0LA                106    0.071  10.913   1.062   0.970   0.001   0.030
B P  2C0LA                107    0.203  28.763   0.068   0.923   0.002   0.076
B S  2C0LA                108    0.145  17.017  -0.649   0.858   0.002   0.139
B K  2C0LA                109    0.106  21.701   0.799   0.782   0.003   0.216
B R  2C0LA                110    0.099  22.671  -2.470   0.858   0.002   0.139
B I  2C0LA                111    0.097  17.908   0.272   0.923   0.002   0.076
B L  2C0LA                112    0.031   5.676  -0.393   0.970   0.001   0.030
E G  2C0LA                113    0.270  21.241   0.514   0.970   0.001   0.030
B S  2C0LA                114    0.159  18.670   0.046   0.970   0.001   0.030
B L  2C0LA                115    0.092  16.772   0.309   0.970   0.001   0.030
E L  2C0LA                116    0.284  51.927   0.368   0.923   0.002   0.076
E S  2C0LA                117    0.670  78.559   0.924   0.782   0.003   0.216
E D  2C0LA                118    0.399  57.481  -0.068   0.246   0.004   0.750
E S  2C0LA                119    0.593  69.546  -0.798   0.109   0.005   0.886
E L  2C0LA                120    0.432  79.172   1.349   0.053   0.005   0.942
B F  2C0LA                121    0.158  31.650   0.937   0.694   0.003   0.303
E L  2C0LA                122    0.724 132.509   1.217   0.938   0.007   0.055
E E  2C0LA                123    0.460  80.345   1.785   0.975   0.003   0.022
B V  2C0LA                124    0.013   1.983   1.290   0.970   0.001   0.030
B K  2C0LA                125    0.175  35.895   1.349   0.970   0.001   0.030
E E  2C0LA                126    0.593 103.527   1.790   0.970   0.001   0.030
B L  2C0LA                127    0.122  22.393   1.263   0.970   0.001   0.030
B F  2C0LA                128    0.031   6.161   0.928   0.970   0.001   0.030
E L  2C0LA                129    0.435  79.649   2.099   0.988   0.000   0.012
E A  2C0LA                130    0.397  43.716   2.171   0.988   0.000   0.012
B A  2C0LA                131    0.021   2.347   1.015   0.970   0.001   0.030
B V  2C0LA                132    0.071  10.943   0.554   0.975   0.003   0.022
E R  2C0LA                133    0.632 144.659   1.634   0.879   0.010   0.111
B L  2C0LA                134    0.208  38.048   1.007   0.600   0.003   0.397
B D  2C0LA                135    0.220  31.702   1.110   0.053   0.005   0.942
E P  2C0LA                136    0.503  71.390  -0.655   0.016   0.005   0.979
E T  2C0LA                137    0.764 105.925   0.351   0.003   0.003   0.994
B S  2C0LA                138    0.293  34.328  -0.313   0.018   0.019   0.964
E I  2C0LA                139    0.447  82.621  -1.367   0.058   0.017   0.925
E D  2C0LA                140    0.341  49.181   0.028   0.115   0.016   0.868
B P  2C0LA                141    0.117  16.673  -1.042   0.923   0.002   0.076
E D  2C0LA                142    0.327  47.193   0.712   0.970   0.001   0.030
B V  2C0LA                143    0.037   5.702   0.205   0.970   0.001   0.030
B Q  2C0LA                144    0.044   7.841   0.692   0.970   0.001   0.030
B C  2C0LA                145    0.112  15.795   0.160   0.970   0.001   0.030
B G  2C0LA                146    0.091   7.146  -0.762   0.923   0.002   0.076
B L  2C0LA                147    0.054   9.814   1.190   0.970   0.001   0.030
B G  2C0LA                148    0.023   1.786  -0.747   0.970   0.001   0.030
B V  2C0LA                149    0.175  26.882   1.235   0.970   0.001   0.030
B L  2C0LA                150    0.024   4.449   0.815   0.988   0.000   0.012
B F  2C0LA                151    0.098  19.649   0.953   0.970   0.001   0.030
E N  2C0LA                152    0.343  50.171   0.692   0.923   0.002   0.076
E L  2C0LA                153    0.533  97.684   1.258   0.858   0.002   0.139
E S  2C0LA                154    0.333  39.028   0.154   0.321   0.003   0.675
E G  2C0LA                155    0.656  51.604  -0.170   0.053   0.005   0.942
E E  2C0LA                156    0.450  78.563   1.355   0.053   0.005   0.942
B Y  2C0LA                157    0.178  37.974   0.931   0.600   0.003   0.397
E D  2C0LA                158    0.714 102.916   1.344   0.938   0.007   0.055
E K  2C0LA                159    0.496 102.109   1.911   0.938   0.007   0.055
B A  2C0LA                160    0.013   1.422   1.274   0.975   0.003   0.022
B V  2C0LA                161    0.155  23.808   1.461   0.970   0.001   0.030
E D  2C0LA                162    0.594  85.639   1.805   0.970   0.001   0.030
B C  2C0LA                163    0.122  17.143   1.319   0.970   0.001   0.030
B F  2C0LA                164    0.030   5.941   0.890   0.988   0.000   0.012
E T  2C0LA                165    0.459  63.622   2.181   0.988   0.000   0.012
E A  2C0LA                166    0.413  45.535   2.335   0.970   0.001   0.030
B A  2C0LA                167    0.024   2.656   0.776   0.970   0.001   0.030
B L  2C0LA                168    0.095  17.358   1.007   0.938   0.007   0.055
E S  2C0LA                169    0.637  74.715   1.567   0.802   0.014   0.185
E V  2C0LA                170    0.244  37.534   1.263   0.522   0.016   0.462
E R  2C0LA                171    0.335  76.830   1.663   0.016   0.005   0.979
E P  2C0LA                172    0.509  72.284  -0.601   0.053   0.005   0.942
E N  2C0LA                173    0.675  98.776   0.006   0.016   0.005   0.979
B D  2C0LA                174    0.156  22.465   0.258   0.053   0.005   0.942
B Y  2C0LA                175    0.207  44.343  -0.136   0.923   0.002   0.076
E L  2C0LA                176    0.463  84.867   1.177   0.970   0.001   0.030
B L  2C0LA                177    0.035   6.427   0.214   0.970   0.001   0.030
B W  2C0LA                178    0.052  12.506   0.829   0.970   0.001   0.030
B N  2C0LA                179    0.141  20.657   0.054   0.970   0.001   0.030
B K  2C0LA                180    0.167  34.311  -0.281   0.970   0.001   0.030
B L  2C0LA                181    0.058  10.565   1.136   0.970   0.001   0.030
B G  2C0LA                182    0.025   1.991  -1.039   0.970   0.001   0.030
B A  2C0LA                183    0.168  18.569   1.039   0.970   0.001   0.030
B T  2C0LA                184    0.028   3.884   0.551   0.988   0.000   0.012
B L  2C0LA                185    0.096  17.559   0.983   0.970   0.001   0.030
E A  2C0LA                186    0.308  33.997   0.680   0.938   0.007   0.055
E N  2C0LA                187    0.546  79.934   1.060   0.858   0.002   0.139
E G  2C0LA                188    0.314  24.696   0.142   0.321   0.003   0.675
E N  2C0LA                189    0.626  91.676  -0.548   0.053   0.005   0.942
E Q  2C0LA                190    0.401  71.565   1.403   0.053   0.005   0.942
B S  2C0LA                191    0.170  19.924   0.777   0.600   0.003   0.397
E E  2C0LA                192    0.670 117.066   1.132   0.938   0.007   0.055
E E  2C0LA                193    0.477  83.419   1.844   0.975   0.003   0.022
B A  2C0LA                194    0.013   1.455   1.246   0.975   0.003   0.022
B V  2C0LA                195    0.152  23.316   1.414   0.970   0.001   0.030
E A  2C0LA                196    0.592  65.282   1.716   0.970   0.001   0.030
B A  2C0LA                197    0.148  16.343   1.668   0.970   0.001   0.030
B Y  2C0LA                198    0.031   6.603   0.805   0.988   0.000   0.012
E R  2C0LA                199    0.459 105.134   2.123   0.988   0.000   0.012
E R  2C0LA                200    0.398  91.073   2.349   0.970   0.001   0.030
B A  2C0LA                201    0.025   2.777   0.742   0.970   0.001   0.030
B L  2C0LA                202    0.109  20.013   0.616   0.938   0.007   0.055
E E  2C0LA                203    0.663 115.861   1.561   0.879   0.010   0.111
E L  2C0LA                204    0.238  43.559   1.001   0.502   0.002   0.495
E Q  2C0LA                205    0.312  55.812   1.338   0.016   0.005   0.979
E P  2C0LA                206    0.460  65.288  -0.653   0.016   0.005   0.979
E G  2C0LA                207    0.691  54.397  -0.021   0.016   0.005   0.979
B Y  2C0LA                208    0.159  34.064   0.599   0.115   0.016   0.868
B I  2C0LA                209    0.192  35.557  -0.267   0.923   0.002   0.076
E R  2C0LA                210    0.436  99.775   1.171   0.970   0.001   0.030
B S  2C0LA                211    0.029   3.364   0.313   0.970   0.001   0.030
B R  2C0LA                212    0.058  13.374   0.770   0.970   0.001   0.030
B Y  2C0LA                213    0.150  32.119  -0.109   0.970   0.001   0.030
B N  2C0LA                214    0.128  18.754  -0.219   0.970   0.001   0.030
B L  2C0LA                215    0.059  10.766   1.129   0.970   0.001   0.030
B G  2C0LA                216    0.028   2.172  -1.358   0.970   0.001   0.030
B I  2C0LA                217    0.200  36.944   1.170   0.970   0.001   0.030
B S  2C0LA                218    0.027   3.176   0.865   0.988   0.000   0.012
B C  2C0LA                219    0.102  14.293   0.984   0.970   0.001   0.030
E I  2C0LA                220    0.373  68.912   1.134   0.938   0.007   0.055
E N  2C0LA                221    0.587  85.922   1.076   0.879   0.010   0.111
E L  2C0LA                222    0.291  53.264   0.858   0.406   0.004   0.590
E G  2C0LA                223    0.650  51.147  -0.167   0.109   0.005   0.886
E A  2C0LA                224    0.377  41.600   1.524   0.053   0.005   0.942
B H  2C0LA                225    0.183  33.306   1.263   0.694   0.003   0.303
E R  2C0LA                226    0.709 162.315   1.250   0.938   0.007   0.055
E E  2C0LA                227    0.461  80.537   1.918   0.938   0.007   0.055
B A  2C0LA                228    0.011   1.223   1.355   0.975   0.003   0.022
B V  2C0LA                229    0.170  26.098   1.268   0.970   0.001   0.030
E E  2C0LA                230    0.606 105.956   1.935   0.970   0.001   0.030
B H  2C0LA                231    0.116  21.028   1.224   0.970   0.001   0.030
B F  2C0LA                232    0.030   6.121   0.905   0.988   0.000   0.012
E L  2C0LA                233    0.470  86.130   2.149   0.988   0.000   0.012
E E  2C0LA                234    0.400  69.967   2.201   0.988   0.000   0.012
B A  2C0LA                235    0.022   2.413   0.935   0.970   0.001   0.030
B L  2C0LA                236    0.103  18.933   0.739   0.938   0.007   0.055
E N  2C0LA                237    0.657  96.229   1.673   0.879   0.010   0.111
B M  2C0LA                238    0.192  38.339   1.137   0.622   0.015   0.363
E Q  2C0LA                239    0.262  46.882   1.506   0.053   0.005   0.942
E R  2C0LA                240    0.507 115.989  -0.477   0.016   0.005   0.979
E K  2C0LA                241    0.734 150.984   0.240   0.016   0.005   0.979
B S  2C0LA                242    0.165  19.303  -0.010   0.053   0.005   0.942
E R  2C0LA                243    0.470 107.653  -0.670   0.858   0.002   0.139
E G  2C0LA                244    0.537  42.223   0.291   0.923   0.002   0.076
B P  2C0LA                245    0.063   8.911  -0.413   0.923   0.002   0.076
B R  2C0LA                246    0.143  32.678   0.054   0.858   0.002   0.139
E G  2C0LA                247    0.308  24.208  -0.572   0.858   0.002   0.139
B E  2C0LA                248    0.256  44.671  -1.399   0.782   0.003   0.216
B G  2C0LA                249    0.121   9.483  -0.122   0.858   0.002   0.139
B G  2C0LA                250    0.116   9.129  -1.791   0.858   0.002   0.139
E A  2C0LA                251    0.289  31.815   0.581   0.923   0.002   0.076
B M  2C0LA                252    0.084  16.828  -0.185   0.923   0.002   0.076
B S  2C0LA                253    0.106  12.458  -0.279   0.923   0.002   0.076
E E  2C0LA                254    0.346  60.481  -0.569   0.970   0.001   0.030
E N  2C0LA                255    0.364  53.290   0.430   0.970   0.001   0.030
B I  2C0LA                256    0.043   7.955  -0.551   0.970   0.001   0.030
B W  2C0LA                257    0.156  37.566  -0.443   0.970   0.001   0.030
E S  2C0LA                258    0.343  40.176  -0.631   0.923   0.002   0.076
B T  2C0LA                259    0.155  21.499  -1.002   0.923   0.002   0.076
B L  2C0LA                260    0.069  12.542   0.116   0.970   0.001   0.030
B R  2C0LA                261    0.129  29.564  -1.250   0.970   0.001   0.030
E L  2C0LA                262    0.289  52.971   1.087   0.970   0.001   0.030
B A  2C0LA                263    0.025   2.799   0.472   0.970   0.001   0.030
B L  2C0LA                264    0.105  19.262   0.407   0.970   0.001   0.030
E S  2C0LA                265    0.475  55.705   0.701   0.923   0.002   0.076
E M  2C0LA                266    0.473  94.707   0.828   0.858   0.002   0.139
B L  2C0LA                267    0.254  46.434   0.381   0.502   0.002   0.495
E G  2C0LA                268    0.629  49.518  -0.222   0.246   0.004   0.750
E Q  2C0LA                269    0.408  72.887   1.136   0.246   0.004   0.750
B S  2C0LA                270    0.175  20.522   0.955   0.782   0.003   0.216
E D  2C0LA                271    0.701 101.043   1.204   0.938   0.007   0.055
E A  2C0LA                272    0.434  47.871   1.786   0.938   0.007   0.055
B Y  2C0LA                273    0.014   2.970   1.180   0.970   0.001   0.030
B G  2C0LA                274    0.218  17.188   1.471   0.970   0.001   0.030
E A  2C0LA                275    0.595  65.536   1.838   0.970   0.001   0.030
B A  2C0LA                276    0.100  11.042   0.888   0.970   0.001   0.030
B D  2C0LA                277    0.042   6.009   0.730   0.970   0.001   0.030
E A  2C0LA                278    0.537  59.210   1.916   0.970   0.001   0.030
E R  2C0LA                279    0.414  94.737   2.192   0.970   0.001   0.030
B D  2C0LA                280    0.027   3.847   0.404   0.970   0.001   0.030
B L  2C0LA                281    0.075  13.787   0.573   0.923   0.002   0.076
E S  2C0LA                282    0.682  79.966   1.549   0.782   0.003   0.216
E T  2C0LA                283    0.274  38.073   1.115   0.502   0.002   0.495
E L  2C0LA                284    0.238  43.505   1.266   0.053   0.005   0.942
E L  2C0LA                285    0.430  78.733   0.384   0.053   0.005   0.942
E T  2C0LA                286    0.778 107.964   0.472   0.053   0.005   0.942
E M  2C0LA                287    0.379  75.758   0.318   0.053   0.005   0.942
B F  2C0LA                288    0.192  38.474   0.155   0.016   0.005   0.979
E G  2C0LA                289    0.623  49.038   0.762   0.016   0.005   0.979
B L  2C0LA                290    0.156  28.582  -0.515   0.016   0.005   0.979
E P  2C0LA                291    0.459  65.118  -0.580   0.018   0.019   0.964
E Q  2C0LA                292    0.803 143.362   0.163   0.003   0.003   0.994

Explain the output.  Go back.
```

---
